# Catatonia in Ugandan children with nodding syndrome and effects of treatment with lorazepam: a pilot study

**DOI:** 10.1186/s13104-015-1805-5

**Published:** 2015-12-28

**Authors:** Angelina Kakooza-Mwesige, Dirk M. Dhossche, Richard Idro, Dickens Akena, Joyce Nalugya, Benard T. Opar

**Affiliations:** Department of Pediatrics and Child Health, Makerere University College of Health Sciences, P O Box 7072, Kampala, Uganda; Neuropaediatric Unit, Department of Women’s and Children’s Health, Karolinska Institute, Stockholm, Sweden; University of Mississippi Medical Center, 2500 North State Street, Jackson, MS 39216 USA; Nuffield Department of Medicine, Centre for Global Health and Tropical Medicine, University of Oxford, Oxford, UK; Department of Psychiatry, Makerere University College of Health Sciences, P.O. Box 7072, Kampala, Uganda; Ministry of Health Headquarters, Kampala, Uganda

**Keywords:** Catatonia, Children, Lorazepam, Nodding syndrome, Uganda

## Abstract

**Background:**

Nodding syndrome (NS) is a severe neuropsychiatric syndrome of an unknown etiology affecting children and adolescents mostly in Eastern Africa. Symptoms of NS and catatonia seem to overlap. We investigated the presence and types of catatonic symptoms in NS and their response to one or two doses of lorazepam, the first-line treatment for catatonia.

**Methods:**

A cross-sectional descriptive study with systematic assessment of catatonia in 33 patients with NS using a modified version of the Bush Francis Catatonia Rating Scale. Sixteen patients met criteria for catatonia and were observed in an open and uncontrolled study to examine the effects of one or two doses of lorazepam in them.

**Results:**

Sixteen of 33 patients with NS had an average of 5 catatonia symptoms and met criteria for catatonia. The highest scores were found for mutism, staring, poor eating/drinking, stupor, and grimacing. Excitement, rigidity, negativism and impulsivity had lower scores. None of the children had echolalia or echopraxia. In 6 children, there was a reduction of more than 50 % in catatonia ratings, representing a positive response to lorazepam. Three out of six children whose catatonia ratings did not change after the first dose, responded after administration of a second double dose. There were no unusual or critical side-effects.

**Conclusions:**

About half of a selected sample of children with NS met criteria for catatonia. Catatonia scores decreased in most patients after one or two doses of lorazepam. Larger, longer, and controlled studies are warranted to assess the prevalence of catatonia in NS and to assess the use of lorazepam in NS through its effects on catatonia.

Trial Registration: ClinicalTrials.gov NCT02462109

Date of formal registration: June 2, 2015

**Electronic supplementary material:**

The online version of this article (doi:10.1186/s13104-015-1805-5) contains supplementary material, which is available to authorized users.

## Background

Nodding syndrome (NS) is a neurological condition of unknown etiology that has affected over 10,000 or more children and adolescents aged 3–18 years in the sub-Saharan countries of Uganda, Tanzania and South Sudan [1–3]. NS is a clinical constellation of symptoms that comprises of head nodding, often associated with cognitive impairment, malnutrition, other multiple seizure types and progressive neurological deterioration [4]. In Uganda, NS has occurred in epidemic proportions especially in the post-conflict northern districts of Lamwo, Kitgum and Pader with over 170 deaths reported [4] and few other cases reported in Amuru, Gulu, Lira and Oyam districts indicating increasing geographical coverage.

Results from a single-stage cluster survey conducted in three districts in northern Uganda among children aged 5–18 years gives an estimated prevalence of 6.8 probable NS cases (95 % CI 5.9–7.7) per 1000 [5]. The hallmark of NS is the ‘head nods’ that have been attributed to atonic seizures [1]. NS is complicated by seizures which may be recurrent and severe generalized tonic–clonic, myoclonic, focal or atypical absence types. Furthermore there is deterioration in social interaction and communication skills coupled with movement and posture activity limitations, malnutrition, and behavioral and emotional disturbances [6]. A number of classifications have been proposed to classify patients with NS, Winkler et al. [2] classified them as either head nodding only or head nodding plus, if they also had other seizure types. At the first international scientific meeting on NS held in Uganda in July 2012 [4], a new consensus case definition was drawn to classify them into suspect, probable or confirmed cases. To date, in addition to an undetermined etiology, there is no diagnostic test and NS lacks a definitive treatment although anti-epileptic drugs, nutritional supplements, antidepressants are thought to improve aspects of the disorder [4].

Catatonia is a unique syndrome characterized by specific motor signs, at times life-threatening when aggravated by autonomic dysfunction and fever, yet treatable if recognized early [7, 8]. The syndrome occurs worldwide including in resource-limited settings and responds well to benzodiazepines as first-line treatment [9, 10]. The syndrome also occurs in children and adolescents [11, 12] with a variety of associated conditions including neurological disorders [13, 14].

Signs observed in pediatric and adult catatonia, regardless of associated medical or neurological condition, include immobility or severe motor slowing, sometimes alternating with excessive motor activity that is mostly purposeless and not influenced by external stimuli, extreme negativism (pervasive refusal to comply with requests including refusal to drink and eat), reduced speech or mutism, repetitive movements or stereotypy, echolalia, echopraxia, and other peculiarities of voluntary movement [15].

Some of the neuropsychiatric features [16] of NS are similar to what is seen in pediatric catatonia. Patients with NS seem to exhibit several catatonic symptoms which range from: the head nods when nodding is viewed as a form of tic, slowing of movements, immobility alternating with purposeless agitation, muteness, repetitive movements, staring, posturing, grimacing, social withdrawal, negativism (including active or passive refusal to eat and drink), and urinary incontinence. Aggressive and self-injurious behavior and hallucinations have also been reported both in children with catatonia and NS. No prior studies have formally assessed the presence of catatonia in NS patients using standardized criteria. It is therefore important to ascertain the presence of catatonia in patients with NS to clarify the neuropsychiatric impairment and to find new therapeutic approaches.

Given the clinical similarities between NS and catatonia, one may postulate that NS may be a form of pediatric catatonia and that circumstances in Northern Uganda and surrounding areas provide a crucible of risk factors, both medical and psychological, leading to catatonic development and impairment [17]. Pediatric catatonia is treatable and very responsive to benzodiazepines and in refractory cases, to electroconvulsive therapy [11, 12, 15], also in cases associated with neurological conditions [13, 14].

A positive catatonia test (i.e., when a 50 % reduction of symptoms follows acute administration of a benzodiazepine) would verify the presence of catatonia and serve as a possible treatment option in this poorly understood neurological disorder.

In this pilot study, we examined a convenient sample of children and adolescents with nodding syndrome for catatonic symptoms using standardized criteria. We also tested whether oral lorazepam (LZP) administered to those who qualified to have pediatric catatonia would alleviate symptoms. We hypothesized that children and adolescents with nodding syndrome meet clinical criteria for pediatric catatonia and children and adolescents with nodding syndrome having symptoms of pediatric catatonia would have a positive response (>50 % reduction of symptom severity) after administration of test doses of Lorazepam.

## Methods

### Study design

This was a cross-sectional descriptive study of catatonia in NS patients in Northern Uganda and an open label pilot study of using one or two doses of lorazepam as a catatonia test.

### Study setting

The study was conducted in Pader District, situated in Northern Uganda (one of the three districts reported to be most affected by NS) in the villages of Bolo-Laming and Lagotoywech. Pader was formerly part of Kitgum District and borders Lamwo to the northwest, Kitgum District to the northeast, Gulu in the west, Agago in the east and Lira and Otuke in the South (see Additional file 1: Figure S1). Pader forms part of the northern Ugandan region that has been the site of a protracted (over 20 years) rebel insurgency that displaced over one million people into internally displaced camps [16]. Following the return of peace the security situation has significantly improved with disbandment of the internally displaced people’s (IDP) camps and most of the population returning to their ancestral homes. The district headquarters at Pader are located approximately 130 km (81 miles), by road, northeast of Gulu, the largest city in the sub-region. The district population is over 481,400 persons. The primary occupation of both men and women following resettlement is agriculture, livestock and small business.

### Study participants

Young people aged 10–21 years, who fulfilled the NS diagnosis according to the consensus definition by international NS researchers [4], who presented with two or more catatonic items from a list of ten preselected common catatonia symptoms coined the Kampala Catatonia Panel (see section on assessment of catatonia) and whose caregivers gave written informed consent, were consecutively recruited. Children without a caregiver to provide the history of the child, a history of having used a benzodiazepine drug in the past 48 h, or having a concurrent acute illness (e.g. febrile illness, pneumonia) at time of assessment were excluded.

### Study definitions

Young people were defined as individuals between ages 10 and 24 years [18].

Epilepsy was defined as recurrence of, at least, two epileptic seizures with or without a positive response to treatment with any anti-epileptic drug [19].

A suspected NS case was defined as reported head nodding (repetitive involuntary drops of the head towards the chest on two or more occasions) in a previously normal person.

A probable NS case was defined as a suspected case with age of onset at 3–18 years and a frequency of nodding of 5–20 nods per minute, plus at least one of six minor criteria.

A confirmed NS case was defined as a probable case with a documented nodding episode that was either observed and recorded by a trained health-care worker, videotaped, or documented with video electroencephalography or electromyography as atonic seizures (See Additional file 2: Table S1).

### Procedure of enrolment and study measurements

In March 2013, AKM travelled to Pader District where a single-stage cluster survey to determine the prevalence of NS in Uganda using the new consensus case definition was being conducted by the Centers for Disease Control (CDC) Atlanta with collaboration from the Ugandan Ministry of Health. The children with probable NS had earlier been identified by village health workers during a house-to-house census conducted in July 2012 in the northern Ugandan district of Pader. During the census, Village health workers had queried the heads of households whether anyone in their households had or had ever had head nodding. All children with probable NS within the district were then mobilized to attend the respective treatment centers during this time. The screening and treatment centers for NS were initially set up in March 2012 in the northern Ugandan districts of Kitgum, Lamwo, and Pader by the Uganda Ministry of Health, where most NS cases have been reported. At these centers the probable NS cases receive medical treatment such as anticonvulsants, nutritional rehabilitation in form of therapeutic feeds, speech and language therapy, physiotherapy, psychosocial support for behavioral problems and appropriate care for any other existing co-morbidities. The details of this treatment have previously been described [6].

Each child with probable NS brought to the treatment center in Bolo-Laming and Lagotoywech were further assessed by staff of the census medical team using a standardized questionnaire to further classify the cases based on the consensus criteria. Medical and family social history, were obtained, anthropometric measurements were taken and the developmental and cognitive function of the child assessed. When the census team had completed their investigations, the children with confirmed NS were screened by the principal investigator (AKM) for possible catatonia using a modified version of the Bush-Francis Catatonia Rating Scale, (BFCRS), a 23-item, standardized instrument for catatonia diagnosis and assessment of severity [20] that is used to measure treatment response [21]. Before obtaining written informed consent from the caregivers AKM, explained to the caregivers via an interpreter the risks and benefits of the procedure.

All consecutive cases who qualified for the inclusion criteria were recruited following written informed consent from the caregiver.

### Assessment for catatonia

When using the BFCRS, catatonia is usually diagnosed when two or more items on the first 14 items are present. Scores on all 23 items are between 0 and 69 and gauge severity. In this study, a more stringent diagnosis of catatonia was used, and patients qualified for a diagnosis of catatonia if 2 or more items of 10 items selected from the 14 BFCRS screening items were present.

These 10 items were selected from the 14 BFCRS screening items as the most important and obvious symptoms of catatonia. Four BFCRS screening items, i.e., waxy flexibility, verbigeration, posturing, and mannerisms were not included as they are less frequently found in catatonic patients [20] and require a more elaborate assessment that was difficult to do in the field setting of this study. Severity was assessed using a Likert-scale of 0–5 (0 = absent, 1 = maybe present; 2 = a little bit; 3 = clearly present; 4 = a lot; 5 = extreme), with a maximum score of 50. The 10-item list used to assess for catatonia in this study formed the Kampala Catatonia Panel (KCP) test and the items selected are listed in Table 1.

**Table 1:** Kampala catatonia panel consisting of ten screening items for catatonia

1	Excitement	Extreme hyperactivity, constant motor unrest which is apparently non-purposeful, includes aggressive episodes
2	Immobility-stupor	Extreme hypoactivity, remains in the same position for hours, immobility. Minimally responsive to stimuli
3	Mutism	Verbally unresponsive or minimally responsive
4	Staring	Fixed gaze, little or no visual scanning of environment, decreased blinking
5	Grimacing	Facials spasms, facial tics, or maintenance of odd facial expressions
6	Echopraxia or echolalia	Mimicking of examiner’s movements/speech
7	Rigidity	Maintenance of a rigid position despite efforts to be moved
8	Negativism	Apparently motiveless resistance to instructions or to attempts to move/examine the patient. Contrary behavior, does the opposite of the instruction
9	Active or passive refusal to eat or drink	Minimal or reduced food and fluid intake during the past week
10	Impulsivity	Patient suddenly engages in inappropriate behavior (e.g., runs down the hallway, starts screaming, or takes off clothes) without provocation. Afterwards, cannot explain

### Sample size

No sample size calculations were employed since this was a pilot study and the number of persons included were a random sample of convenience comprising a proportion of all the patients that had been confirmed to have NS by the census team at these respective centers. The two selected villages made up part of a parish (a parish contains multiple villages). The census team selected 30 parishes by single-stage cluster sampling with probability proportional to size from the northern Ugandan districts of Kitgum, Lamwo, and Pader, where most NS cases have been reported. In each parish 20–30 children with reported head nodding were selected using simple random sampling without replacement and invited with their caregivers to a central meeting point for verification.

### The intervention

All the children with confirmed NS that had two or more of the symptoms on the KCP were recruited by AKM to undergo the catatonia test using oral Lorazepam EG^®^ (n.v. Eurogenerics s.a. Brussels, Belgium) using the 1 mg formulation tablets. It was proposed to perform a catatonia test using LZP as first choice medication, as this is the medication that has been used most commonly in pediatric catatonia.

The amount of drug given was based on the weight of the child. The lower dose (0.5 mg) was used as starting dose for patients with <30 kg body weight, while the higher dose (1 mg) as the starting dose for patients with >30 kg body weight.

A positive response to a catatonia test consisted of a reduction in catatonic symptoms, 30 or 60 min later, by at least 50 % assessed by the KCP (using all 10 items). Positive responses were documented by video footage before and after administration of LZP.

If no response to the initial dose of LZP, was observed after 1 h, a second administration of the same medication at double the dose was given by AKM. Catatonia was again assessed by AKM at 30 and 60 min thereafter. If no response was observed, the test was considered negative.

### Primary outcome measures

Our primary outcomes were: (a) the proportion of children and adolescents with NS who met clinical criteria for pediatric catatonia; and (b) a positive catatonia test (>50 % reduction in catatonia symptoms and signs) on the responses of the children and adolescents with NS and catatonia to test doses of oral LZP.

### Ethical considerations

Approval of the study was granted by the School of Medicine Research and Ethics Committee, Makerere University College of Health Sciences and the Uganda National Council of Sciences and Technology (Reference HS 1330). Written informed consent was obtained from the caregivers/parents. No assent was obtained from the children in view of their cognitive challenges.

### Data analysis

Descriptive characteristics of the group of young people with NS and pediatric catatonia were analyzed according to socio-demographic characteristics, catatonia symptoms, lorazepam dosage and outcome. Results are summarized as frequencies, proportions and means/medians as appropriate. Microsoft Excel for Mac 2011 software was used for analysis.

## Results

### Description of study participants

**Fig. 1:**
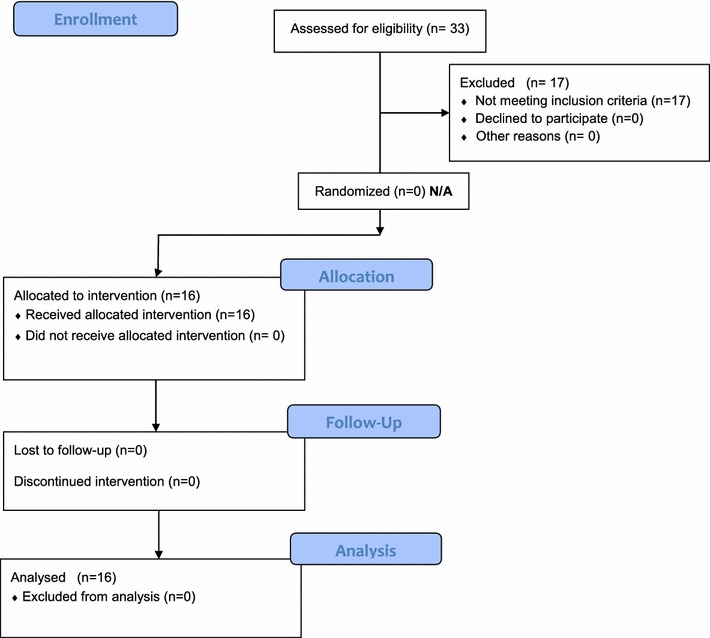
Flowchart showing the recruitment and assessment of the study participants with catatonia

A total of 33 consecutive patients with confirmed NS from the villages of Bolo-Laming and Lagotoywech in Pader district were screened for catatonia over a two-day period. For details see Fig. 1. Sixteen children out of 33 (48.5 %) had 2 or more catatonic symptoms on the KCP fulfilling the criteria for catatonia. Ten children were identified in the village of Bolo-Laming village and six in the village of Lagotoywech. There were 8 female and 8 male NS children identified with Catatonia.

Age range was between 10 and 21 years with a mean age of 15 years (SD 2.95) and a mode and median age of 15 years. Two children were in school, seven dropped out, and seven never went to school. Four children carried a diagnosis of NS (without seizures) (see Table 2). Twelve children with NS had additional symptoms such as seizures or regression in growth or mental retardation. Ten children were taking sodium valproate and six children carbamazepine. None of the children were febrile or had taken benzodiazepines during the last 2 days.

**Table 2:** Summary table showing the socio-demographic characteristics, catatonia symptoms, LZP dosage and outcome of the sampled NS children

Child ID	Age (years)	BMI	WHO-BMI Classification	School attendance	Case classification	Current drug treatment	Catatonia symptoms before treatment	Initial catatonia score	Initial dose of LZP (mg)	Score 60′ after first dose	Repeat dose of LZP	Score 60′ after second dose	Outcome	Side effects
CT001	15	16.44	Moderate thinness	Never	Head nodding syndrome plus	Sodium valproate	2, 3, 4, 5, 9	17	1	4	None		Improved	None
CT002	18	18.66	Underweight	Dropped out P.4	Head nodding syndrome	Sodium valproate	2, 3, 4, 7, 9	19	1	9	None		Improved	Sedation Seizure (fell down)
CT003	15	15.98	Severe thinness	Never	Head nodding syndrome plus	Sodium valproate	1, 3, 4, 5, 7, 8, 10	22	1	2	None		Improved	None
CT004	15	12.55	Severe thinness	Dropped out P.3	Head nodding syndrome plus	Sodium valproate	2, 3, 4, 5, 9	17	0.5	10	None		Improved	Focal seizure with automatisms
CT005	14	14.81	Severe thinness	Never	Head nodding syndrome plus	Sodium valproate	2, 3, 4, 5, 7, 8, 9	24	1	24	2 mg	4	Improved	None
CT006	13	15.18	Severe thinness	Dropped out P.3	Head nodding syndrome	Carbama-zepine	1, 2, 3, 4, 9	15	1	15	2 mg	15	No improvement	Sedation
CT007	10	13.48	Severe thinness	In school in P.1	Head nodding syndrome	Carbama-zepine	3, 4, 5	10	0.5	10	1 mg	1	Improved	None
CT008	10	12.07	Severe thinness	Never	Head nodding syndrome plus	Sodium valproate	2, 3, 4, 5, 8, 9	17	0.5	6	None		Improved	None
CT009	14	20.48	Normal	Dropped out P.1	Head nodding syndrome plus	Sodium valproate	2, 4, 5, 9	8	1	8	2 mg	14	No improvement Nodding episode seen	Sedation Seizure (fell down)
CT010	15	18.65	Normal	In school in P.3, demoted from P.6	Head nodding syndrome	Sodium valproate	2, 3, 4, 7, 9	12	1	12	2 mg	12	No improvement	Sedation
CT011	16	17.5	Mild thinness	Never	Head nodding syndrome plus	Carbama-zepine	1, 3, 4, 5, 8, 9, 10	27	1	27	2 mg	9	Improved	Sedation
CT012	19	15.06	Severe thinness	Dropped out P.1	Head nodding syndrome plus	Sodium valproate	2, 3, 4, 5, 7, 9	20	1	12	None		Improved	Sedation
CT013	18	13.43	Severe thinness	Never	Head nodding syndrome plus	Sodium valproate	2, 3, 4, 5, 7, 8, 9	25	0.5	9	None		Improved	Sedation
CT014	13	10.36	Severe thinness	Never	Head nodding syndrome plus	Carbama-zepine	2, 3, 4, 5, 7, 9	20	0.5	12	None		Improved	Sedation
CT015	21	16.3	Moderate thinness	Dropped out P.1	Head nodding syndrome plus	Carbama-zepine	1, 2, 3, 4, 7, 9, 10	27	1	13	None		Improved	Drooling saliva
CT016	15	14.1	Severe thinness	Dropped out P.2	Head nodding syndrome plus	Carbama-zepine	1, 2, 3, 4, 5, 7, 9	18	1	7	None		Improved	None

Key for the catatonia symptoms	
Number	Symptom
1	Excitement
2	Immobility-stupor
3	Mutism
4	Staring
5	Grimacing
6	Echopraxia or echolalia
7	Rigidity
8	Negativism
9	Active or passive refusal to eat or drink
10	Impulsivity

### Evaluation for catatonia

Using the KCP 10-item list, the mean number of symptoms (with severity score of 2 or higher) per child was 5.4 (range 2–8, SD 1.5). The highest scores were found for mutism, staring, poor eating/drinking, stupor, and grimacing. Excitement, rigidity, negativism and impulsivity had lower scores. None of the children had echolalia or echopraxia.

The number of children that received scores three or higher—indicating that symptoms were clearly present—was 16 for staring, 12 for mutism, 11 for poor eating/drinking, 6 for both stupor and grimacing, 3 for both excitement and impulsivity, 2 for negativism, nine for rigidity, and none for echolalia/echopraxia.

### Drug administration of lorazepam

Twenty-two doses of LZP were administered; all 16 children received an initial dose of 0.5 mg or 1 mg, depending on their weight. Six children received an increased second dose (1 or 2 mg) after 60 min when no changes in catatonia ratings had occurred in the previous hour.

### Effects of lorazepam

**Fig. 2:**
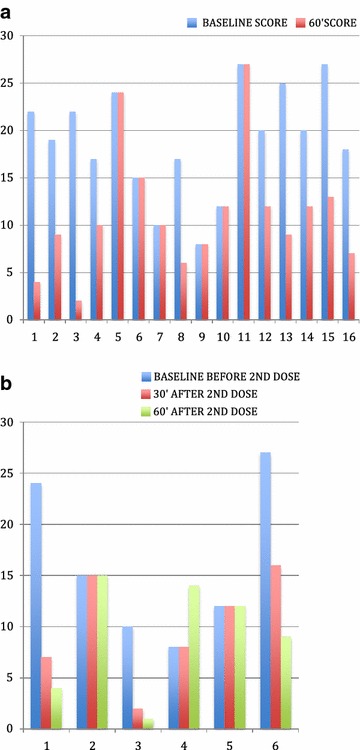
**a** Graph showing changes in catatonia scores with first dose of treatment after 60 min. **b** Graph showing the changes in catatonia scores with second dose of treatment after 30 and 60 min

In 11 children (>30 kg), the initial dose was 1 mg and in 5 children (<30 kg) it was 0.5 mg. The changes in catatonia scores from baseline to 60 min after administration and the percent of reduction of catatonia scores are shown in Fig. 2a.

Following the first dose, in 6 children there was a reduction of more than 50 % in catatonia ratings, representing a positive response. In 4 children there was a reduction in catatonia symptoms, however they did not reach the ratings of a positive catatonia test. Catatonia ratings decreased an average of 57 % in those 10 children whose catatonia ratings decreased. In 6 children, scores did not change and a repeat dose was given (see Fig. 2a, b).

In 5 children a repeat dose of 2 mg was given, and in one child the repeat dose was 1 mg. Scores at baseline and after 30′ and 60′ after the second dose are shown in Fig. 2b. Three children improved, two remained the same, and catatonia ratings increased in one child.

### Side effects of the intervention

Focal seizures were observed in three children (CT002, CT004, and CT009) after administration of the dose of LZP. These seizures were clinically observed after the first dose in two children (CT002, CT004) and involved either one side of the lips and/or upper and lower limbs. No electroencephalographic equipment was available to verify these findings.

One child (CT009) became restless after administration of the 2 mg repeat dose, followed by a nodding episode. She then became drowsy and unresponsive. Her catatonia rating increased due to increased stupor that was probably related to seizure activity.

Four children had some sedation after the first dose. Four of the five children who were administered increased repeat doses had some sedation after 60′ of administration and some fell asleep later (see Table 2).

## Discussion

This investigation was the first to attempt to formally assess for catatonia in NS. About half of a selected sample of children with NS that were screened for catatonia had two or more symptoms of catatonia and qualified for the diagnosis of catatonia. A mean number of 5 catatonic symptoms (range 2–8, SD 1.5) was found in those 16 patients, most frequently mutism, staring, poor eating/drinking, stupor, and grimacing. A few had excitement and showed impulsive behavior. None had echolalia or echopraxia. This finding is in line with previous studies showing that catatonic patients have usually more symptoms than the two symptoms that are needed to qualify for the diagnosis [20]. These findings raise the issue of shared elements in the psychopathology of NS and catatonia, and maybe in their aetiology and pathophysiology as well [17]. The clinical manifestations of NS also show resemblance with those of epilepsy, late-onset autism spectrum disorder, or early-onset schizophrenia.

Head nodding is considered the hallmark of NS. Some patients develop recurrent generalized tonic–clonic or focal seizures. The head nods are thought to be manifestations of atonic seizures [1, 2, 22] but may also be viewed as a form of tic or stereotypy, a common autistic [23] and catatonic symptom [24]. The course of NS consists of a period of normal development, followed by onset of repetitive movements including head nodding, decreasing social interaction & communication skills, and decline in intelligence & daily activities, similar as in late-onset autism. The later onset of NS and period of normal development preclude a diagnosis of classic autism (that starts before or around age 3) yet the social withdrawal, speech and communication impairment, and repetitive movements are compatible with autism spectrum disorder [25]. In addition autistic and catatonic symptoms overlap [15, 26] and treatments that ameliorate catatonia seem to benefit patients who meet both criteria for autism spectrum disorders and onset of catatonia [27].

Some children with NS report hallucinations and become aggressive and/or self-injurious leaving a heavy burden on the family and caretakers [16]. Hallucinations and behavioral problems are the hallmark of early-onset schizophrenia, another disorder that shows clinical overlap with NS. Catatonic symptoms are thought to be common in early-onset schizophrenia [28]. There are currently no studies that have systematically assessed symptoms of schizophrenia or autism spectrum disorder in patients with NS.

The high occurrence of seizures in NS has prompted the notion that NS is a primary progressive epileptic disorder (of unknown origin) [1] yet not all NS cases have documented seizure activity and not all clinical features of NS may be linked to primary epilepsy [29]. In addition, the use of anti-epileptic drugs seems beneficial but not curative. It would be premature to dismiss clinical phenomena such as catatonia or psychosis that do not easily fit the scope of primary progressive epilepsy especially as the aetiology and pathophysiology of NS remain obscure. Leads that NS is associated with current or prior infection with *Onchocera volvulus*, vitamin B6 or other vitamin deficiencies need further study [30].

This investigation was also the first to assess the acute effects of single or repeated lorazepam treatment on NS. Catatonia ratings decreased in 10 of 16 patients after the first dose of LZP, with 6 patients having a score reduction of more than 50 % and being formal responders. Such a positive challenge test supports the presence of catatonia in the study subjects as it is known that catatonia is exquisitely responsive to benzodiazepines [21, 31].

Alternative explanations are that any improvements in NS by lorazepam or other benzodiazepines are more related to their anxiolytic or antiepileptic properties than their anti-catatonic effects. However, the effects of lorazepam and other benzodiazepines on catatonia may be linked through their anxiolytic and antiepileptic effects as extreme anxiety and subthreshold epileptic activity are among the putative mechanisms of catatonia [32, 33]. In addition, Musisi et al. [16, 34] have called attention to the association between NS and post-traumatic stress disorder as many children with NS have suffered considerable war-related and other interpersonal trauma. Anxiety- and stress-related symptoms in NS may respond to benzodiazepines in addition to psychomotor features of NS. Teasing out the effects of lorazepam on anxiety, epileptic activity, and the motor symptoms of catatonia will require further studies. Regardless of the mechanism, findings of this pilot study beg the question whether treatment with lorazepam or another benzodiazepine in NS can have an important role in improving overall impairment in NS both in its acute and chronic stages.

### Limitations

First, this was a feasibility study using a small number of patients hence the results should be interpreted with caution. Secondly, there is a potential that the NS cases were misclassified by the trained health worker as video-EEG/EMG was not available in all cases. Thirdly, since the children were mobilized to attend the treatment center by the village health workers, there is a potential of nonresponse bias which may have excluded NS patients with catatonia who were unable to walk or travel to the treatment center. Finally, the LZP trial was open and uncontrolled obscuring any placebo effects and/or any biases of non-blind assessments.

## Conclusions

The findings from this pilot study suggest that a significant proportion of children with NS, at least in this selected sample, meets criteria for catatonia, and that single and repeated administration of LZP is safe and may be beneficial in NS. Acute but transient improvement of catatonia after administration of LZP supports and validates that catatonia is present, encouraging larger and longer studies of catatonia and LZP in NS management.

Larger, longer, and controlled studies are needed to verify the prevalence of catatonia in NS and to assess if repeated doses of LZP in NS sustain the clinical improvements that are apparent after single doses of LZP. A next step is to systematically assess catatonia in a larger sample of children and adolescents with NS, to initiate LZP in those who meet criteria for catatonia, to optimize the dose and administer LZP twice a day, and to continue treatment for 4–6 weeks. Ideally, an untreated control group would be enrolled using clinical catatonia assessments done in a blind fashion.

## Additional files

10.1186/s13104-015-1805-5 Map of Uganda showing Pader district.
10.1186/s13104-015-1805-5 Consensus case definition for Nodding Syndrome - Uganda, 2012.

## Abbreviations

BFCRS: Bush Francis Catatonia Rating Scale
CDC: Centres for Disease Control
EEG: electroencephalography
EMG: electromyogram
KCP: Kampala Catatonia Panel
NS: nodding syndrome
LZP: lorazepam
