# Supplementary figures and images for: Catatonia in Ugandan children with nodding syndrome and effects of treatment with lorazepam: a pilot study
Source: BMC Res Notes. 2015 Dec 28;8:825. doi: 10.1186/s13104-015-1805-5 (PMC4693437; doi:10.1186/s13104-015-1805-5)

**Supplementary Figure 1: Map of Uganda showing Pader district.**

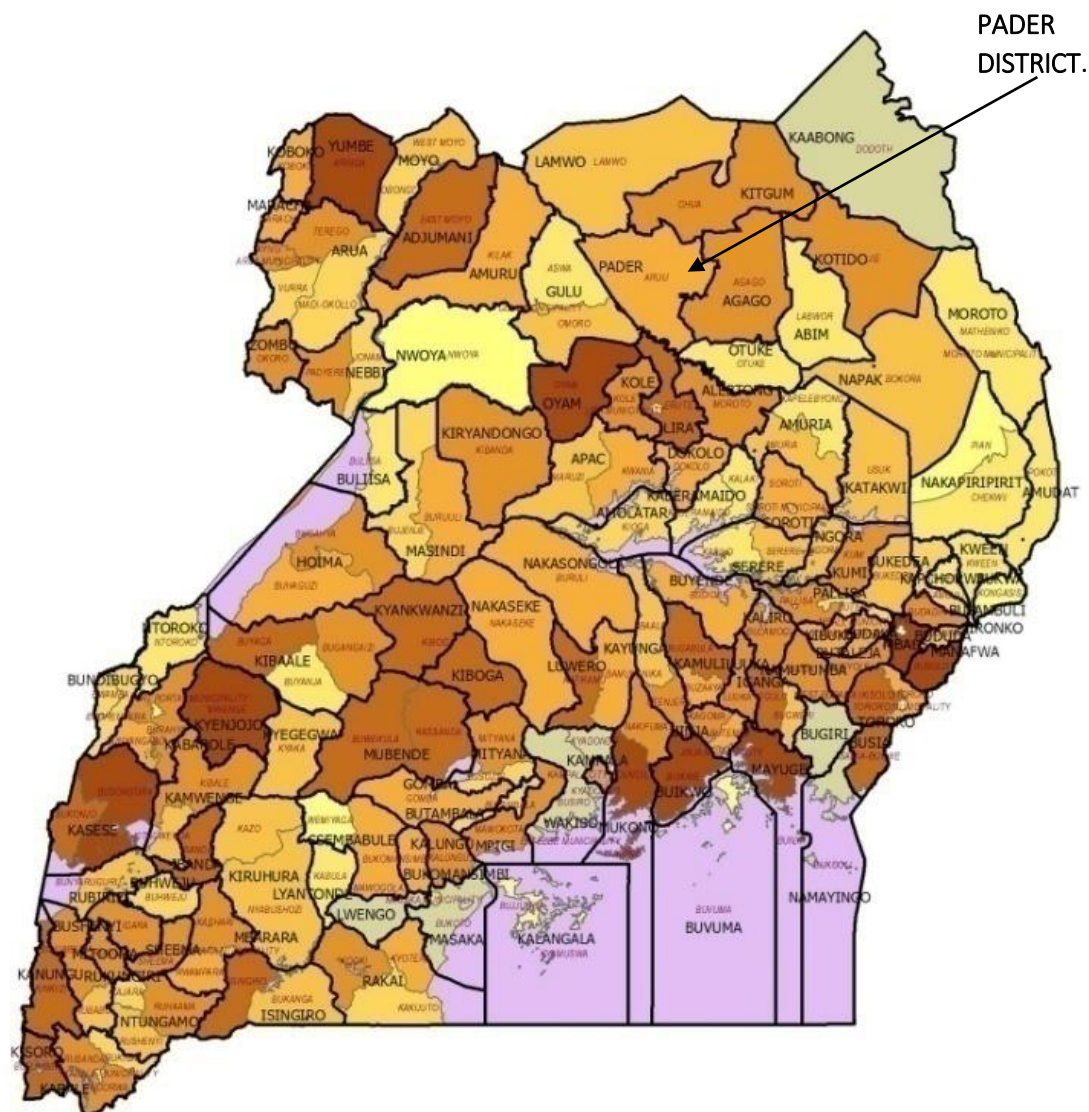

Supplement: Supplementary file 1 — 10.1186/s13104-015-1805-5 Map of Uganda showing Pader district. [file 13104_2015_1805_MOESM1_ESM.pdf]
