# Supplementary material for: Catatonia in Ugandan children with nodding syndrome and effects of treatment with lorazepam: a pilot study
Source: BMC Res Notes. 2015 Dec 28;8:825. doi: 10.1186/s13104-015-1805-5 (PMC4693437; doi:10.1186/s13104-015-1805-5)
Supplement: Supplementary file 2 — 10.1186/s13104-015-1805-5 Consensus case definition for Nodding Syndrome - Uganda, 2012. [file 13104_2015_1805_MOESM2_ESM.pdf]

Supplementary Table 1: Consensus case definition for nodding syndrome - Uganda, 2012[#]

Type of case	Consensus case definition
**Suspected case**	Reported head nodding (repetitive involuntary drops of the head towards the chest on two or more occasions) in a previously normal person
**Probable case**	Suspected case of head nodding, with both major criteria:<br>• Age of onset of nodding ranging from 3 to 18 years<br>• Frequency of nodding 5–20 per minute
	Plus at least one of the following minor criteria:<br>• Other neurologic abnormalities (cognitive decline, school dropout because of cognitive or behavioral problems, other seizures or neurologic abnormalities)<br>• Clustering in space or time with similar cases<br>• Triggering by food or cold weather<br>• Stunting or wasting<br>• Delayed sexual or physical development<br>• Psychiatric symptoms
**Confirmed case**	Probable case, with documented nodding episode<br>• Observed and recorded by a trained health-care worker, or<br>• Videotaped nodding episode, or<br>• Video/EEG/EMG documenting head nodding as atonic seizures

Abbreviations: EEG = electroencephalographic; EMG = electromyographic.

[#]The consensus case definition was drafted at the first International Scientific Meeting on Nodding Syndrome, held July 30–August 1, 2012, in Kampala, Uganda. Meeting report available at http://www.who.int/neglected_diseases/diseases/Nodding_syndrom_Kampala_Report_2012.pdf
